# Heterogeneous Tapped Delay-Line Time-to-Digital Converter on Artix-7 FPGA

**DOI:** 10.3390/s25092923

**Published:** 2025-05-06

**Authors:** Riguang Chen, Ping Chen, Kuinian Li, Hulin Liu

**Affiliations:** 1 Key Laboratory of Ultra-Fast Photoelectric Diagnostics Technology, Xi’an Institute of Optics and Precision Mechanics of CAS, Xi’an 710119, China; chenriguang22@mails.ucas.ac.cn (R.C.); likuinian@opt.ac.cn (K.L.); hulin@opt.ac.cn (H.L.); 2 University of Chinese Academy of Sciences, Beijing 100049, China; 3 Collaborative Innovation Center of Extreme Optics, Shanxi University, Taiyuan 030006, China

**Keywords:** Time-to-Digital Converter (TDC), Field-Programmable Gate Array (FPGA), Tapped Delay Line (TDL), time-of-flight (TOF) measurements, time interval (TI) measurement

## Abstract

Time-to-Digital Converters (TDCs) implemented on Field-Programmable Gate Arrays (FPGAs) have become increasingly prevalent across a wide range of scientific and engineering disciplines, such as high-energy physics experiments, autonomous driving, robotic navigation, and medical imaging, owing to their cost-effectiveness, high precision, and rapid development cycles. This article presents a 3-tap heterogeneous tapped delay-line (TDL) architecture for a FPGA-based TDC that can be employed for multi-channel time-of-flight measurement. The TDC desgin is based on the open-source jTDC, featuring single-cycle dead time and multi-channel expansion capabilities, with an original precision of 30 ps. Combined with jTDC’s dynamic caching mechanism using dual-page memory, this work employs a dual-cycle encoding and calibration. The proposed architecture has been implemented on a Xilinx Artix-7 FPGA. According to the experimental results, an optimal 3-tap heterogeneous TDL architecture achieves a resolution of 23.220 ps and a typical precision of 17.520 ps, whereas an optimal 4-tap heterogeneous TDL architecture demonstrates a resolution of 17.530 ps and a typical precision of 17.213 ps. A comparison with recently published state-of-the-art FPGA-based TDCs is provided at the end of the article.

## 1. Introduction

Analogous to how an Analog-to-Digital Converter (ADC) quantizes signal amplitude into digital representation, a Time-to-Digital Converter (TDC) digitizes time intervals between two pulses. Both converters share fundamental performance metrics [1,2], including resolution, dynamic range, Differential Nonlinearity (DNL), Integral Nonlinearity (INL), and dead time. For TDCs specifically, resolution denotes the minimum measurable time interval, while practical measurement precision is typically characterized by the standard deviation of repeated time-interval measurements.

High-precision TDCs are predominantly implemented through Field-Programmable Gate Arrays (FPGAs) or Application-Specific Integrated Circuits (ASICs). FPGA-based TDCs offer distinct advantages in flexibility, rapid deployment, and lifecycle cost efficiency, making them particularly suitable for applications with evolving requirements, tight development schedules, or limited production scales. Their implementation spans multiple scientific and engineering domains requiring precise temporal measurements:

- In high-energy physics experiments like those at the Large Hadron Collider [3], FPGA-TDCs paired with microchannel plate photomultipliers (MCP-PMTs) enable precise particle arrival time measurements for trajectory reconstruction and energy distribution analysis, leveraging their high precision and real-time processing capabilities.
- LiDAR systems in autonomous vehicles [4,5] and robotic navigation [6] employ FPGA-TDCs to enhance spatial resolution and system reliability through improved time-of-flight measurements.
- Medical imaging modalities such as Positron Emission Tomography (PET) benefit from FPGA-TDCs’ superior time resolution and multi-channel capabilities for accurate photon arrival time detection [7,8].

The broad application prospects of FPGA-TDCs have driven their rapid development, resulting in multiple mature technical approaches [9]. These TDCs generally adopt a coarse-fine two-stage quantization architecture: the coarse counting module achieves rough time measurements by accumulating clock cycles, while the fine counting module uses techniques like Tapped Delay Lines (TDLs) or Phased Clocks [10] for subcycle interpolation within the clock period. The resolution of the phased clock scheme depends on the phase difference between the sampling clocks. It requires the generation of multiple phase-shifted clocks through a mixed-mode clock manager (MMCM) or a phase-locked loop (PLL) to achieve interpolation. However, the number of interpolations is constrained by the number of MMCMs or PLLs and their output ports. In contrast, the TDL scheme can achieve stable resolution by implementing specific delay units and easily attain picosecond-level measurement accuracy. Therefore, current high-precision and ultra-high-precision TDCs primarily adopt TDL or TDL hybrid architectures. The TDL scheme has become a main research direction due to its simplicity and high precision. To improve the resolution and accuracy of TDLs, in addition to calibrating bin widths of TDLs, multichain TDLs can be used for differential or averaging processing. To balance precision and resource utilization, Wu and Shi [11] proposed the wave-union (WU) method, which improves TDL resolution without increasing the number of delay lines. Won and Lee [12], based on the actual structure of Xilinx FPGA carry chains, proposed a Tuned-TDL heterogeneous structrue, to improve TDL linearity and accuracy. Parsakordasiabi et al. [13], building on the Tuned-TDL concept, introduced a dual-mode design that further balances high precision and low resource utilization, achieving a Single-shot Precision (SSP) of 22.35 ps on Artix-7.

The Tuned-TDL methodology specifically addresses non-uniform sampling intervals in carry chains by strategically combining Carry (C) and XOR (S) outputs (e.g., SSSS, CCCC, SCSC), thereby enhancing linearity and measurement accuracy. Based on the open-source jTDC by BIELING John [14], this work integrates Tuned-TDL principles to achieve a precision of 17.213 ps. Implemented on the Ailinx AX7203 development board (XC7A200T-2FBG484 FPGA), the proposed TDC operates with a sampling clock frequency of 500 MHz, enabling shorter delay chains. Furthermore, we introduce a novel 3-tap CSC heterogeneous delay chain configuration that achieves a precision of 17.520 ps. While this performance is inferior to optimal 4-tap heterogeneous architecture, it represents an effective improvement over conventional delay chains, which exhibit a precision of 17.998 ps.

The rest of the article is organized as follows. The basic working principal of an TDL-TDC and the proposed heterogeneous TDL-TDC is described in Section 2. The design methodology of the jTDC and the architecture of the heterogeneous TDL are explained in detail. Experimental measurement results are presented in Section 3, which also includes detailed explanations of the experimental setup, calibration methodology, evaluation criteria and a comparative analysis with single TDL-TDCs from recent years. Finally, Section 4 summarizes the key features of the proposed TDC and concludes the article.

## 2. Design of the Proposed TDC

### 2.1. Basic Priciple of TDL-TDC

The implementation scheme of a TDC is selected based on the target resolution requirements. In conventional application scenarios (>2 ns), a basic architecture based on clock counters can meet measurement needs, where the time resolution corresponds to the system clock period. However, when measurement accuracy requirements increase to sub-nanosecond or even picosecond levels, traditional counter approaches would require reference clocks operating at tens of GHz frequencies, posing significant challenges even when implemented in ASICs. Therefore, to achieve high-precision measurement of fine time intervals, the adoption of interpolation sampling methods becomes an essential complementary approach.

**Figure 1:**
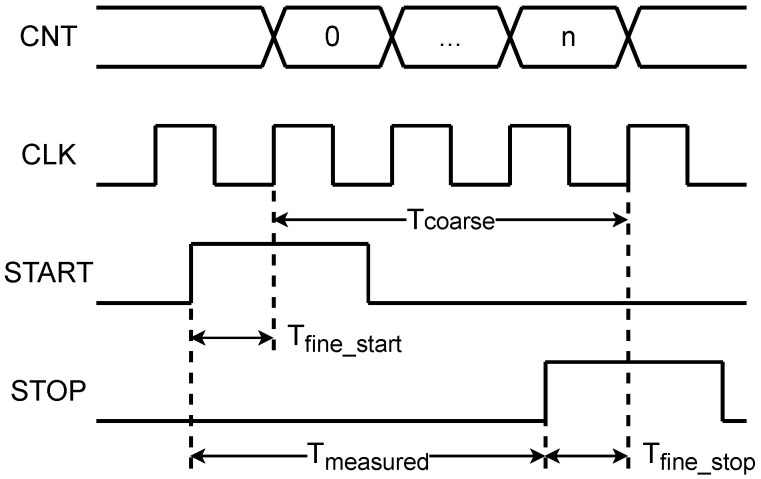
Schematic diagram of the measurement principle combining coarse and fine counting.

For the measurement of the time interval between a pair of START/STOP signals, as shown in Figure 1, the coarse counting uses a clock counter to calculate the number of cycles *n* between the START and STOP signals. Combined with the sampling clock period $T_{\mathrm{CLK}}$, this yields the coarse time $T_{\mathrm{coarse}}=n\cdot T_{\mathrm{CLK}}$. Fine counting, on the other hand, employs interpolation sampling via a delay line and sampling encoding to capture the time differences $T_{\mathrm{fine\_start}}$ and $T_{\mathrm{fine\_stop}}$ between the signal edges and the sampling clock edges. The final measured value $T_{\mathrm{measured}}$ is then derived from these results

(1) $$T_{measured}=n\cdot T_{CLK}+T_{fine\text{\_}start}-T_{fine\text{\_}stop}$$

**Figure 2:**
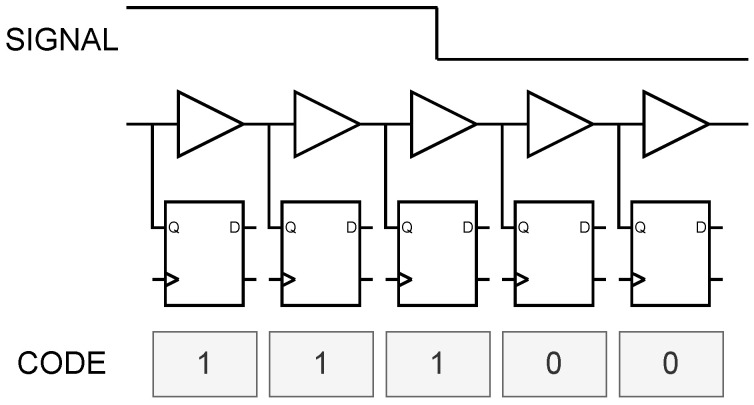
Schematic diagram of TDL sampling structure.

When implementing a high-precision TDC with coarse-fine two-stage quantization on an FPGA platform, the fine counting module typically achieves sub-cycle time interpolation through TDL or phased clock scheme. The working principle of TDL is illustrated in Figure 2: multiple tap points with relatively fixed delay intervals are set along the delay chain. As signals propagate through delay elements, the states of these tap points are recorded at the positive edge of sampling clock. By analyzing corresponding thermometer code, the position of signal edges within the delay chain can be determined, thereby identifying the signal arrival time. In FPGA implementations, delay chains are typically constructed using logic elements (primarily carry chains) or routing resources, which exhibit relatively stable delay characteristics.

The delay characteristics of logic elements inside FPGAs are susceptible to chip process, voltage, and temperature (PVT) variations, which may lead to measurement instability. To address this, calibration techniques such as the Code Density Test (CDT) must be employed to determine the actual delay time of each delay element. Secondly, due to the non-uniformity of FPGA routing resources, delays may vary across different paths. Therefore, optimizing placement and routing becomes crucial during synthesis and implementations to ensure consistency of delay elements. Additionally, when signal edges occur near tap points of the delay chain, metastability in register may be triggered, affecting measurement accuracy. Finally, considering that a single delay chain typically requires hundreds of delay elements, multi-channel integration may consume substantial programmable resources, posing challenges for improving integration density and reducing costs. In practical designs, multiple critical factors including delay uniformity, resolution, calibration methods, and resource constraints must be comprehensively considered to achieve high-performance time-to-digital conversion.

### 2.2. TDC Architecture

**Figure 3:**
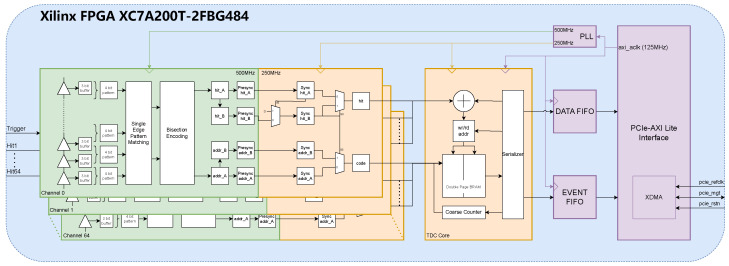
Block diagram of proposed multi-channel TDC.

The proposed FPGA-TDC, as shown in Figure 3, is a 66-channel heterogeneous delay chain TDC based on the open-source jTDC. The jTDC provided an important foundation and design concepts for the development and verification of the digital system in this research. The jTDC is a multi-channel TDC open-sourced by BIELING John [14] from the University of Bonn, featuring a design precision of 30 ps and scalability up to 98 channels, while integrating both pulse counting and time-to-digital conversion functionalities. The 98 extendable channels of jTDC are divided into three groups of 32 LVCMOS33 standard channels each, plus two NIM standard channels serving as trigger inputs, forming a 96 + 2 channel configuration. Being built on jTDC, the proposed TDC implements a design utilizing 64 sampling channels and two trigger channels (64 + 2 channels), while maintaining scalability. Both sampling and trigger channels employ the LVCMOS33 input standard.

To achieve efficient data transmission with the host computer, the proposed TDC employs the Xilinx IP core XDMA to construct a PCIe communication interface. The memory-mapped operations from the host computer are translated into address operations on the on-chip AXI bus via the XDMA IP core, enabling read/write access to the TDC control registers and data FIFOs. Data acquisition is implemented in Ubuntu through a program developed based on XDMA driver.

The internal architecture of the TDC can be divided into three clock domains. To ensure sufficient timing margin for the cross-clock-domain logic, the three clock frequencies maintain integer multiple relationships. The system employs the 125 MHz AXI bus clock provided by the XDMA IP core as the reference clock source, which is frequency-multiplied and synchronized through a PLL to generate two operational clocks at 250 MHz and 500 MHz. The reference clock primarily drives the read/write operations of:

- The Data FIFO caching timing data
- The Event FIFO caching event numbers and event sizes
- The TDC control registers(reset, channel enable, etc.)

The 250 MHz clock domain primarily consists of the TDC core module, which employs a dual-page block RAM memory to enable uninterrupted writing of sampled data (with dead time of one clock cycle), while also performing packet processing and serializing for multi-channel data.

In the 500 MHz clock domain, there are two critical components: the sampling module and the encoding module. Thermometer code received from the carry chain sampler is fed into the encoding module. The encoding module adopts a binary search strategy that prioritizes the first leading edge, encodes the thermometer code into 7 bits, representing the position of the leading edge of the signal inside the chain, which is the desired high resolution time information. The 7-bit information from the encoder is transferred to the 250 MHz clock domain and the state of the 500 MHz clock is stored in an additional 8th bit. Since the sampling clock frequency is twice that of the buffer clock, the encoding module prioritizes the first trigger between two consecutive sampling periods, so that it effectively prevents duplicate triggering caused by delay chains slightly exceeding the sampling period.

### 2.3. Heterogeneous Tapped Delay-Line

**Figure 4:**
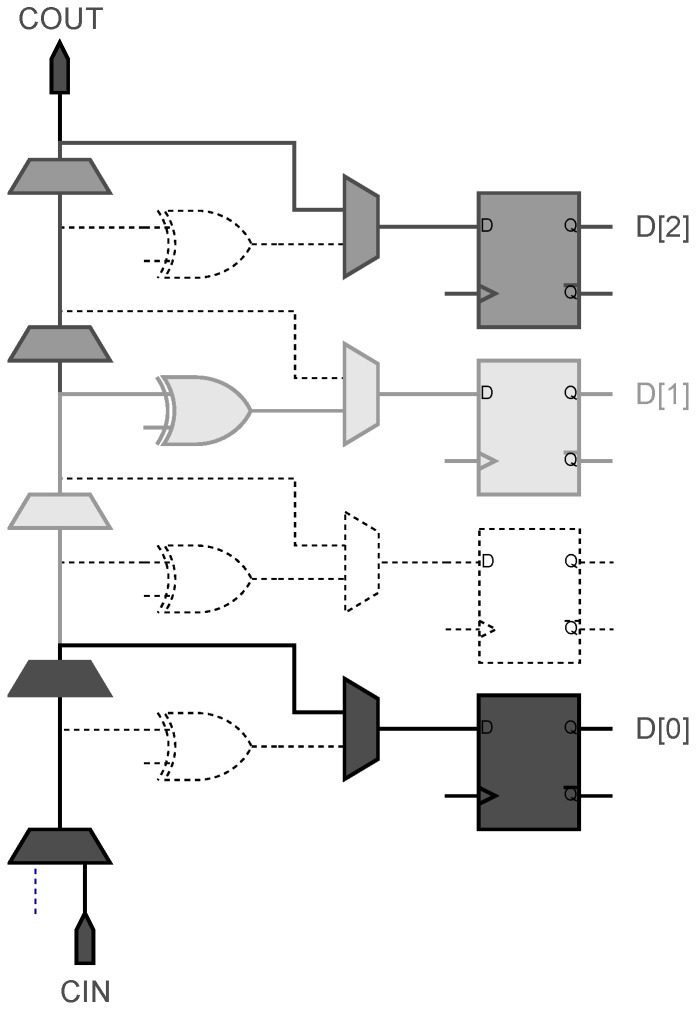
Schematic diagram of a 3-tap CSC carry chain.

In FPGA implementations, TDLs commonly utilize cascaded structures composed of carry chains, Look-Up Tables (LUTs) [15,16], or Digital Signal Processing (DSP) blocks [17], with carry chains being the predominant implementation approach. Within Xilinx 7-series FPGA architectures, programmable logic resources are organized into Configurable Logic Blocks (CLBs), each containing two slices. These slices can be dynamically configured to serve as multipliers, distributed RAMs, or carry chains based on application requirements. Vertical cascading between slices is achieved through dedicated interconnect resources. When configured as carry chains, the schematic diagram of its structure is depicted in Figure 4. Specifically, the carry chain implementation leverages dedicated cascade in (CIN) and cascade out (COUT) ports for vertical cascading. Signal taps can be extracted from the carry out (C) through multiplexers to associated flip-flops, or alternatively routed through XOR (S) gates prior to multiplexer selection. Although each slice contains eight flip-flops, the shared multiplexer architecture between XOR (S) and carry (C) outputs limits each slice to a maximum of four configurable taps, with each tap selectable between carry or XOR outputs.

During signal propagation through the carry chain, traversal of five LUT stages occurs. However, the maximum four-tap constraint per carry chain introduces inherent non-uniformity in unit delay when constructing TDLs, which compromises measurement precision. To mitigate this limitation, heterogeneous delay chain architectures implement differentiated output configurations across taps, effectively equalizing relative delays between tap points and thereby enhancing TDC measurement accuracy.

However, the 4-tap heterogeneous delay chain configuration still exhibits significant numbers of empty or low-delay bins. Considering the relatively stable propagation delay characteristics of cascaded carry chains with optimized lengths, implementing a 3-tap heterogeneous chain configuration enables effective consolidation of empty or low-delay bins. Building upon this principle, our research proposes a novel 3-tap heterogeneous CSC delay chain architecture designed to enhance both uniformity and precision in TDL implementations. As illustrated in Figure 4, distinct gray-scale paths represent signal propagation trajectories of equivalent bins. In this CSC configuration, the most significant bit D[2] and least significant bit D[0] utilize direct carry (C) outputs, while the intermediate bit D[1] employs XOR (S) gate outputs for optimized timing distribution.

### 2.4. Dual Page Memory

**Figure 5:**
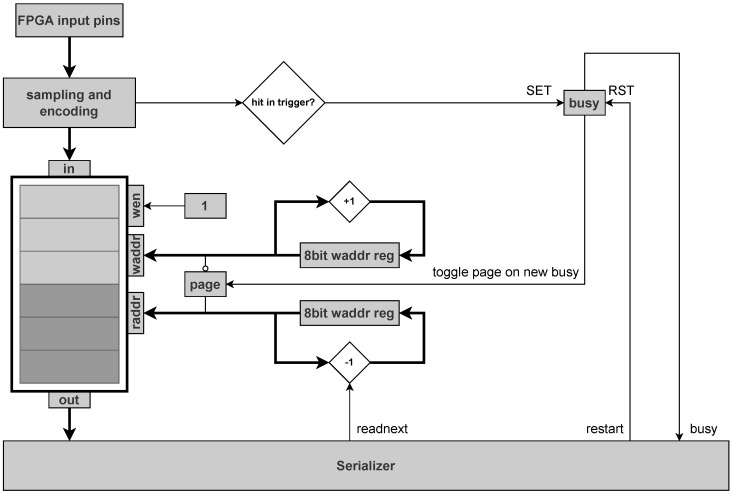
Schematic diagram of double page BRAM memory.

The proposed FPGA-TDC employs identical data processing methodology to jTDC, with its core functionality leveraging FPGA Block RAM (BRAM) resources. Capitalizing on the abundant BRAM capacity inherent to modern FPGAs. As illustrated in Figure 5, the input data of each channel is directly stored in the BRAM without doing any buffering, filtering or sorting. To record data even during read-out, the TDC use a second memory page. On trigger input, the recording is not stopped but simply continued in that second memory page by flipping a page bit. The former write-to-page can be switched to become the read-from-page by using the page bit as the 9th bit of the BRAM read address and the inverted page bit as the 9th bit of the BRAM write address. To prevent further page flipping until the read-from-page has been read-out completely, the page bit is locked until a “restart” command is send. For each input channel, the BRAM has to store the single hit bit (hit or no hit) and the 8bit high resolution time information.

**Figure 6:**
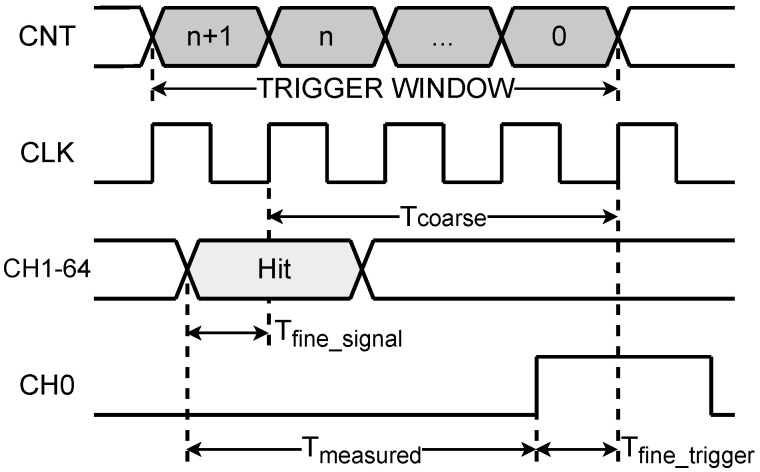
Timing diagram of proposed TDC.

As depicted in Figure 6, the operational timing diagram of proposed TDC diverge from fundamental principles due to its double page memory design. Hit events detected by the encoder module are written to BRAM. When a valid trigger signal arrives, BRAM page-switching occurs while the serial readout module subsequently retrieves chronologically backward both the trigger event and correlated hit events within given trigger window. This operational paradigm necessitates that measured pulses precede trigger pulses in actual measurements, with the resultant time interval being registered as negative offsets relative to the trigger-defined temporal origin.

## 3. Results and Discussion

### 3.1. Calibration

Under ideal conditions where the time intervals between tap points in the delay chain exhibit uniform distribution, the raw TDC encoding directly reflects optimal measurement results. However, due to the inherent non-uniform characteristics of FPGA carry chains and variations in PVT conditions, actual delays between tap points inevitably demonstrate discrepancies. To achieve higher measurement precision, the code density method with bin-by-bin calibration is typically employed to precisely determine the actual delay of each bin. Effective implementation of this method have two critical requirements: First, a sufficiently large sample size must be obtained to ensure statistical accuracy; Second, the input signal must maintain no correlation with the sampling clock, exhibiting uniform distribution characteristics relative to it.

Leveraging the dual page memory and encoding characteristics, the proposed TDC implements code density-based dual-period offline calibration. Through statistical analysis across all samples, the maximum value *N* extracted from the lower 7-bit encoding reveals an effective tap count of N+1 for the delay chain within sampling period *T*. By truncating redundant taps from single-cycle encodings and concatenating dual-cycle codes—where the last tap of the preceding cycle merges with the first tap of the subsequent cycle—the system constructs a 2N+1 tap delay chain (k=0,1,…,2N). Histogram statistics are then collected using a total of Ω samples. Through calculating sample counts n(k) per tap, corresponding delay values t(k) can be estimated to achieve precise delay chain calibration. Code density calibration methods estimate unit delays from tap sample counts. Under uniform distribution assumptions, the fine time measurement t(k) for code *k* can be approximated using trapezoidal integration as (t(0)=0):

(2) $$t\left(k\right)=2T\frac{\int_{0}^{k}n\left(i\right)di}{\int_{0}^{2N}n\left(i\right)di}=\frac{2T}{\Omega }\int_{0}^{k}n\left(i\right)di\approx \frac{T}{\Omega }\sum_{i=1}^{k}\left(n\left(i-1\right)+n\left(i\right)\right),\;k=1,2,\cdots ,2N$$

### 3.2. Characteristics

The resolution of TDC is typically characterized by the time delay $\omega_{LSB}$ corresponding to the Least Significant Bit (LSB). For TDL-based TDCs, $\omega_{LSB}$ is determined by the average time delay per unit in the delay chain. Within a sampling period *T*, where the delay chain with N+1 effective taps corresponds to *N* delay units, the TDC resolution $\omega_{LSB}$ can be expressed as:

(3) $$\omega_{LSB}=\frac{T}{N}$$

The delay chain, composed of multiple delay units, exhibits variations in the time delay $\omega_{k}$ of each units due to layout variations, process variations, and operating conditions. To quantify such deviations, differential nonlinearity (DNL) and integral nonlinearity (INL) are standard metrics employed to characterize the linearity of TDCs.

(4) $$DNL_{k}=\frac{\omega_{k}-\omega_{LSB}}{\omega_{LSB}},k=1,2,\cdots ,N$$

(5) $$INL_{k}=\sum_{i=1}^{k}DNL_{i},k=1,2,\cdots ,N$$

After calibration, the actual time delay of each delay unit can be reconstructed from the time difference between adjacent taps:

(6) $$\omega_{k}=t_{k}-t_{k-1},k=1,2,\cdots ,N$$

For the measurement of a given time interval, the SSP of a TDC can be estimated by its sample standard deviation σ. For a dataset with a sample size of Ω, the calculation formula for the sample standard deviation is:

(7) $$\sigma =\frac{1}{\sqrt{\Omega -1}}\sqrt{\sum_{k=1}^{\Omega }{\left(t_{k}-\;\frac{\sum_{j=1}^{\Omega }t_{j}}{\Omega }\right)}^{2}}$$

### 3.3. Experiment Setup

**Figure 7:**
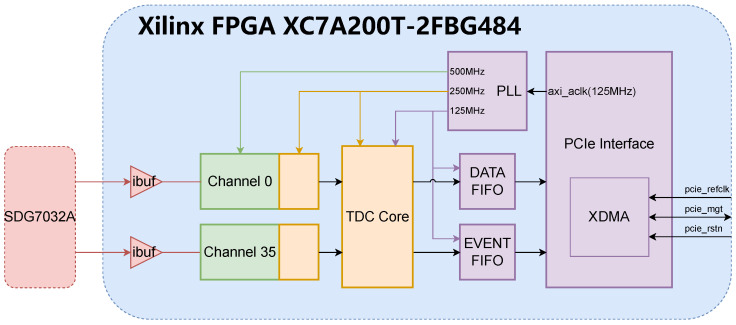
Experiment Setup for testing proposed FPGA-TDC.

To verify the performance of proposed TDC with different TDL configurations, the test setup shown in Figure 7 was implemented. The input signal was generated by an Siglent SDG7032A signal generator operating in pulse mode, with pulse parameters listed in Table 1. By adjusting the signal delay of the Channel 0, a series of reference time intervals were obtained. With dual-cycle calibration, the equivalent duration of a TDL takes two sampling intervals (2 ns), correspond to one complete buffer cycle (4 ns). In order to estimate the characteristics of specific TDL in a complete buffer cycle, a comprehensive time interval sweep was implemented, ranging from 0.25 ns to 4 ns with a 0.25 ns incrementation. The experimental configuration employed an 8 ns sampling window, equivalent to two buffer cycles, ensuring reliable capture of pulse pairs per trigger event with sufficient temporal margin.

For signal input to the FPGA, a dedicated signal transmission circuit board was designed, featuring SMA connectors and coaxial cables for generator connection. Coaxial cables used in the experiment are identical to minimize delay offset of different sampling paths. The circuit board receives coaxial signals, which are then routed to the FPGA development board through board-to-board connectors. To ensure better consistency across different TDL designs, xdc physical constraints were applied to fix the starting points of sampling channels at specific locations, with each channel separated by two rows of CLBs. The strategy “performance_auto_1” was adopted during Vivado implementation.

### 3.4. Results

The experimental evaluation encompassed three different TDL architectures:

- a conventional 4-tap CCCC structure.
- a heterogeneous 4-tap SCSC configuration.
- the proposed 3-tap CSC architecture.

**Figure 8:**
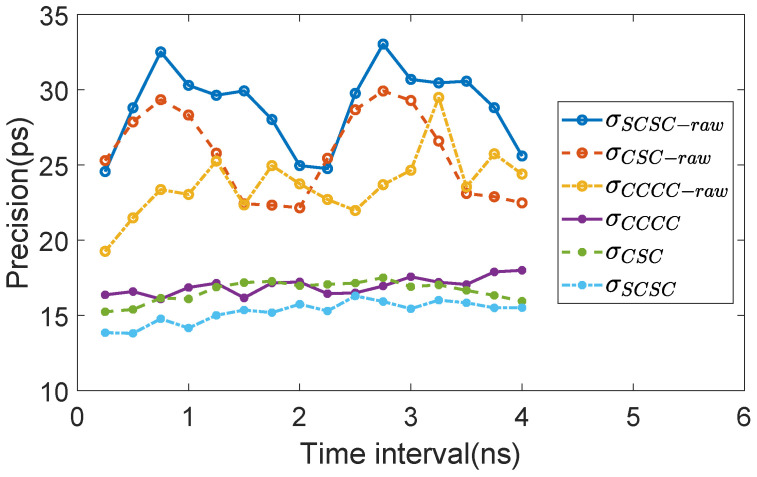
Raw and calibrated precision of different TDL structure.

**Figure 9:**
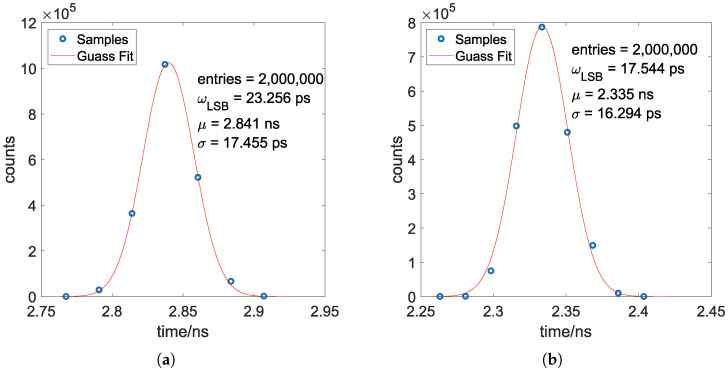
Calibrated measurement histograms of the worst case of SCSC and CSC configuration. (**a**) Calibrated measurement histograms of 3-tap CSC configuration. (**b**) Calibrated measurement histograms of 4-tap SCSC configuration.

The power and resource utilization of various TDL architectures is quantified in Table 2. Notably, due to the substantial resource consumption associated with the XDMA IP Core, the per-channel analysis focuses exclusively on the sampler and encoder modules. Leveraging the high-speed data transfer capability of the PCIe interface, each experimental measurement captures a statistically significant dataset of 2,000,000 samples per time interval, ensuring robust characterization of the temporal response characteristics. The precision of each measurement are calculated with both raw codes and calibrated codes. The results are plotted in Figure 8. The statistical characteristics of each plot are listed in Table 3. Calibrated measurement histograms of the worse case of SCSC and CSC configuration are demonstrated in Figure 9.

It should be noted that the precision result σ presented encompass the cumulative contributions from the entire signal processing chain. Specifically, this includes two primary components, the delay jitter $\sigma_{0}$ origins from the signal generator Siglent SDG7032A and the jitter $\sigma_{\mathrm{TDC}}$ of the proposed TDC itself. According to the datasheet of the signal generator Siglent SDG7032A, its duty cycle resolution of pulses is 0.001%. Accordingly, the delay jitter $\sigma_{0}$ for a 10 MHz pulse signal is then obtained by $\sigma_{0}=0.001\%\times 100\;\mathrm{ns}=1\;\mathrm{ps}$, which is negligible. In conclusion, the jitter $\sigma_{\mathrm{TDC}}$ of the proposed TDC can be effectively approximated by the presented result.

(8) $$\sigma_{\mathrm{TDC}}=\sqrt{\sigma^{2}-\sigma_{0}^{2}}\approx \sigma$$

According to the experimental results, the following information can be obtained:

- CDT calibration demonstrates consistent precision enhancement in TDCs, irrespective of the specific TDL architecture employed. An improvement of over 10 ps for all architectures is obtained. While all precision measurements of the raw codes exceed one LSB, the CDT calibration successfully reduces them to sub-LSB levels.
- Raw code precision does not directly correlate with calibrated code accuracy. Experimental data reveal a phenomenon where architecture exhibiting superior raw code precision demonstrate degraded calibrated precision.
- The 3-tap CSC TDL architecture, featuring a larger $\omega_{LSB}$ compared to 4-tap designs, achieves intermediate performance benchmarks in both raw and calibrated results.
- The 4-tap SCSC TDL architecture demonstrates significant precision enhancement in its calibrated operation, establishing clear advantages over other configurations.

**Figure 10:**
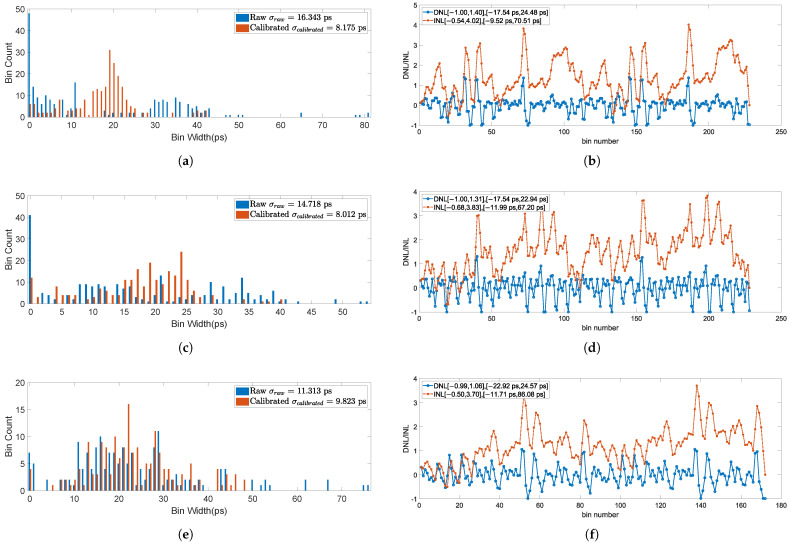
Statistic Analysis of bin width for different TDL configurations. (**a**) Bin width distribution of 4-tap CCCC configuration. (**b**) Calibrated linearity of 4-tap CCCC configuration. (**c**) Bin width distribution of 4-tap SCSC configuration. (**d**) Calibrated linearity of 4-tap SCSC configuration. (**e**) Bin width distribution of 3-tap CSC configuration. (**f**) Calibrated linearity of 3-tap CSC configuration.

To analyze the impact of configuration and calibration on precision metrics, Figure 10 presents the bin width distribution of different configurations along with its calibrated linearity characteristics. It is noteworthy that the linearity assessment was conducted utilizing the complete dataset for a single channel, specifically comprising 16× 2,000,000 samples from channel 35 for each experimental configuration. The findings demonstrated by the data can be summarized as:

- The bin width distribution of the raw code exhibit significant stochastic characteristics, manifesting a considerable proportion of empty bins. Nevertheless, the CDT calibration procedure successfully mitigates both the empty bin phenomenon and the occurrence of excessively large bins, resulting in a well-concentrated distribution centered around $\omega_{LSB}$.
- The raw code bin width distribution of the 3-tap configuration demonstrates significantly superior concentration characteristics compared to its 4-tap counterpart, exhibiting a markedly reduced incidence of empty bins. The calibration process demonstrates a comparatively limited impact on the centralization of the bin distribution, as the raw data inherently exhibits a well-concentrated distribution pattern. Nevertheless, the presence of anomalously large bins persists, which are subsequently effectively addressed through the calibration process.
- All configurations exhibit comparable linearity patterns, with the 3-tap configuration demonstrating optimal linearity performance among the tested variants.

**Figure 11:**
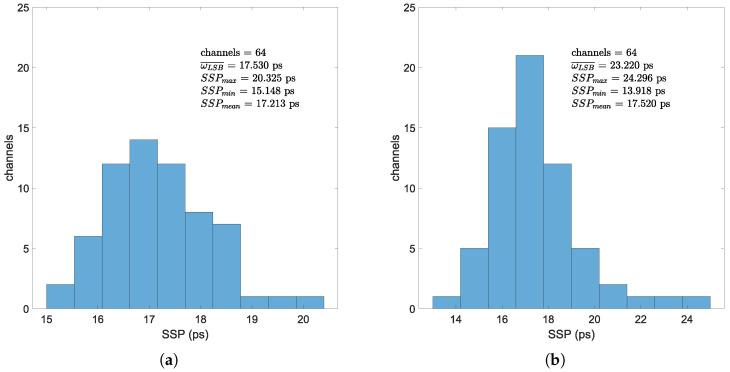
Histogram of multi-channel test result. (**a**) Multi-channel test result of 4-tap SCSC implementation. (**b**) Multi-channel test result of 3-tap CSC implementation.

To evaluate the timing performance of all channels, we independently measured the timing accuracy of all 64 channels in both the 4-tap SCSC TDL and 3-tap CSC implementations, using the same configuration as illustrated in Figure 7. The histogram of multi-channel test results is presented in Figure 11. It is noteworthy that these test results capture inter-channel performance variations, the time-interval-dependent characteristics of the TDLs and potential crosstalk interference between trigger and test channels, primarily arising from electromagnetic coupling effects in PCB trace routing and interface components [18]. While the signal generator maintains a constant 5 ns interval between test and trigger signals, systematic variations in measured time intervals persist due to: (1) significant PCB trace length discrepancies across channels, (2) timing skew introduced during FPGA placement and routing.

### 3.5. Discussion

The experimental result indicates that 3-tap CSC configuration is able to acheive a more linear TDL, with much fewer empty bins and a more concentrated bin width distribution. However, the improved linearity of the TDL does not translate into enhanced calibrated precision. Using the same calibration procedure, the underlying drawbacks can be inferred from the experimental results:

- A 3-tap configuration come with a larger $\omega_{LSB}$ which necessitates significantly improved linearity to compensate. However, the actual enhancement in linearity is demonstrated to be limited. Therefore, the nonlinearity improves by number of LSB, but not by number of picoseconds due larger bin width.
- The 3-tap configuration fails to fully eliminate excessively large bins. Although it exhibits a smaller standard deviation in bin width distribution compared to the 4-tap SCSC configuration, the overall bin width span remains significantly larger, with several oversized bins still present.

From the current perspective, the 4-tap SCSC TDL has been established as the optimal TDL architecture. While the 3-tap CSC TDL falls short of its 4-tap heterogeneous counterpart, it still represents an effective improvement over the conventional 4-tap TDL.

A comparison with recently reported FPGA-based TDL TDCs is provided in Table 4. Except for a few channels with significant deviations, the proposed TDC achieves superior resolution and precision compared to prior work [13,19] on the same FPGA platform. The contributions can be summarized as a higher sampling frequency, a specialized implementation strategy, a robust experimental setup, an optimized calibration method and an edge-matching encoder inherited from jTDC.

## 4. Conclusions

With the aim of eliminating bubbles in the TDL and enhancing linearity, this work proposes a 3-tap heterogeneous CSC TDL architecture for FPGA-based TDC. The proposed TDL architecture has been implemented and validated alongside two other representative 4-tap architectures. The proposed 3-tap architecture has been demonstrated to effectively enhance TDL linearity and exhibits superior precision compared to conventional 4-tap CCCC TDL architecture. And it exhibits the lowest resource consumption and power dissipation. However, certain limitations persist, rendering it less effective than the optimal 4-tap heterogeneous TDL. Based on the open-source jTDC framework, this work have designed and implemented a new TDC on Artix-7 using commercial low-cost development board. In developing this TDC, we modified the jTDC framework to operate at higher clock frequencies while implementing a PCIe communication interface using the XMDA IP core for host system configuration and data acquisition. In optimizing this TDC, we introduced a 4-tap SCSC TDL architecture based on the Tuned-TDL approach, while also proposing a novel 3-tap CSC TDL structure. The experimental results demonstrate a typical precision of 17.213 ps for the 4-tap SCSC TDL and 17.520 ps for the proposed 3-tap TDL. Their resolutions are 17.530 ps and 23.220 ps, respectively.

## Figures and Tables

**Table 1:** Pulse parameters for generating reference signal.

Channel	Frequency	Amplitude	Bias	Pulse Width	Rise Time
0	10 MHz	3 V	1.65 V	5 ns	0.8 ns
35	10 MHz	3 V	1.65 V	5 ns	0.8 ns

**Table 2:** The power and resource utilization of various TDL architectures.

TDL	LUT	FF	Registers per Channel	Slices LUT per Channel	Power per Channel
CCCC	33662	66676	679	271	23 mW
SCSC	37944	66664	679	336	23 mW
CSC	32857	62800	620	259	21 mW

**Table 3:** The statistical characteristics of the precision Results.

	SCSC-Raw	CSC-Raw	CCCC-Raw	SCSC	CSC	CCCC
maximum	33.029	29.916	29.916	16.294	17.506	17.998
minumum	24.561	24.561	22.149	13.808	15.242	16.086
average	24.561	24.561	22.149	15.232	16.610	16.950
$\omega_{LSB}$	17.544	23.256	17.699	17.544	23.256	17.699

The unit is ps.

**Table 4:** Comparison with recently reported FPGA-based TDL TDCs.

Year/Work	Device	LSB (ps)	Precision (ps)	DNL (LSB)	INL (LSB)
This Work	Artix-7	17.530	17.213	[−1.000, 1.307]	[−0.683, 3.830]
		23.220	17.520	[−0.986, 1.057]	[−0.503, 3.702]
2023 [20]	Cyclone-V	5.98	7.6	[−1.00, 4.45]	[−1.71, 2.85]
2022 [13]	Artix-7	22.1	22.35	[−0.71, 1.05]	[−0.85, 0.86]
				[−0.73, 1.06]	[−1.17, 0.04]
2022 [21]	ZYNQ-7020	17.4	19	[−0.90, 1.67]	[−1.90, 3.31]
2022 [22]	Ultrascale	2.48	3.36	[−0.93, 1.68]	[−1.78, 2.67]
2021 [19]	Artix-7	22.2	26.04	[−2.750, 1.238]	[−0.953, 1.185]
2021 [23]	Virtex-6	5.50	6.69	[−0.84, 1.67]	[−3.48, 3.33]
	Kintex-7	1.29	3.54	[−1.20, 1.40]	[−3.28, 3.78]
	Ultrascale	3.95	5.55	[−2.75, 3.00]	[−5.75, 6.00]
2021 [24]	Virtex-6	9	6.2	[−0.90, 3.66]	[−4.74, 26.01]
2019 [25]	Virtex-7	2.03	2.8	[−0.89, 6.20]	[−3.20, 22.30]
2016 [12]	kintex-7	10.6	8.13	[−1.00, 1.45]	[−1.23, 4.30]
	Virtex-6	10.1	9.82	[−1.00, 1.18]	[−3.03, 2.46]
	Spartan-6	16.7	12.75	[−1.00, 1.22]	[−0.70, 2.54]

## Data Availability

The data presented in this study are available on request. The data not contained in the article are not publicly available due to on-going result protection and technology transference processes.
